# Functionalized peptide hydrogels: enabling dynamic stage-adaptive modulation for wound healing

**DOI:** 10.3389/fcell.2025.1710175

**Published:** 2025-11-12

**Authors:** Xi-Kun Ma, Qi Peng, Gui-Hua Miao, Xiu-Zhen Zhang

**Affiliations:** 1 Department of Orthopedic Surgery and Orthopedic Research Institute, West China Hospital, Sichuan University, Chengdu, Sichuan, China; 2 Stem Cell and Tissue Engineering Research Center, State Key Laboratory of Biotherapy, West China Hospital, Sichuan University, Chengdu, Sichuan, China

**Keywords:** functionalized peptide hydrogels, dynamic regulation, multistage targeting, tissue regeneration, wound healing

## Abstract

Functionalized peptide hydrogels represent an emerging class of intelligent wound dressings that dynamically coordinate the multifaceted process of wound healing through stage-specific bioactivities. By leveraging programmable molecular designs, these hydrogels actively engage with key healing phases—hemostasis, antibiosis, inflammation resolution, angiogenesis, and tissue remodeling—enabling spatiotemporal control over cellular behaviors and molecular cues. For instance, antimicrobial peptides (e.g., EPL, LL37, TCP-25) not only eradicate pathogens but also modulate macrophage polarization to mitigate excessive inflammation. Angiogenic factors (e.g., VEGF, SDF-1) are sustainably released to promote vascularization, while MMP-responsive components facilitate ECM remodeling by balancing collagen deposition and degradation. Additionally, integrin-binding motifs (e.g., RGD) enhance cell adhesion and migration, further accelerating re-epithelialization. With inherent self-healing, injectability, and microenvironmental responsiveness (e.g., to pH, enzymes, or ROS), these hydrogels adapt to dynamic wound conditions, offering synergistic therapeutic outcomes. Future directions include designing multi-stimuli-responsive systems, personalized peptide sequences, and advanced delivery platforms (e.g., 3D-printed scaffolds, cell/exosome-loaded hydrogels) to advance clinical translation in precision wound management and regenerative medicine.

## 1 Introduction

**FIGURE 1:**
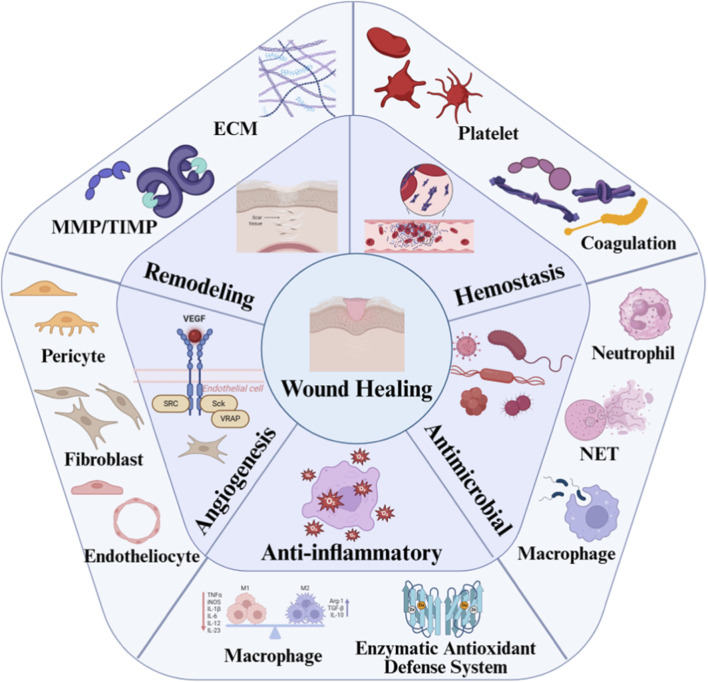
Multicellular involvement across the stages of wound healing.

Wound healing constitutes a highly coordinated, multicellular process encompassing five critical phases: hemostasis, antibiosis, inflammation, angiogenesis, and tissue remodeling (Peña and Martin, 2024). During the hemostatic phase, platelets adhere to the exposed subendothelial matrix via surface integrins (such as αIIbβ_3_ and α_2_β_1_) to form a platelet plug, and release growth factors including Platelet-Derived Growth Factor (PDGF) and Transforming Growth Factor-Beta (TGF-β) (Golebiewska and Poole, 2015). Simultaneously, the coagulation cascade is activated to generate a fibrin network, collectively establishing a provisional matrix (de Bont et al., 2019). They eliminate pathogens through the release of antimicrobial granules (e.g., elastase, cathepsin G), formation of neutrophil extracellular traps (NETs), and phagocytosis (Chen et al., 2018; Sollberger et al., 2018). The inflammatory phase is primarily orchestrated by macrophages. Initially, M_1_-type macrophages secrete pro-inflammatory cytokines such as Tumor Necrosis Factor-Alpha (TNF-α) and Interleukin-6 (IL-6), and phagocytose pathogens. These subsequently transition to M_2_-type macrophages, which promote angiogenesis via vascular endothelial growth factor (VEGF) secretion and resolve inflammation through efferocytosis of apoptotic neutrophils (Murray, 2017; Van Dyken and Locksley, 2013). During angiogenesis, endothelial cells—guided by factors such as VEGF—drive new vessel formation. Pericytes and macrophages provide structural support and paracrine signaling, facilitating the assembly of a functional vascular network (Eilken and Adams, 2010). Finally, in the remodeling phase, myofibroblasts mediate wound contraction through α-smooth muscle actin (α-SMA) expression and secrete extracellular matrix (ECM) components, before being cleared via apoptosis. Macrophages contribute to fibrinolytic activity and remove excess matrix, while the balance between matrix metalloproteinases (MMPs) and tissue inhibitors of metalloproteinases (TIMPs) regulates collagen remodeling, ultimately leading to tissue repair and scar formation (Eilken and Adams, 2010). This multicellular process is finely regulated, and dysregulation at any step may result in impaired healing (Figure 1).

## 2 Functionalized peptide hydrogel

In recent years, functionalized peptide hydrogels have emerged as advanced wound dressings demonstrating significant potential in promoting the healing of both acute and chronic wounds (Shaw et al., 2025). Certain antimicrobial peptides (AMPs), such as ε-polylysine (EPL), GL13L, LLKKK18, and TCP-25, not only exert antibacterial effects by directly disrupting bacterial membrane structures (Liang et al., 2021), but also mitigate excessive inflammatory responses by modulating macrophage polarization—such as promoting the M2 phenotype—thereby reducing pro-inflammatory cytokines (e.g., TNF-α, IL-6) and upregulating reparative cytokines (e.g., TGF-β, IL-10) to achieve phase-appropriate antibacterial and anti-inflammatory effects (Zhou et al., 2023). In terms of angiogenesis, peptide hydrogels can be loaded with and sustainably release VEGF, stromal cell-derived factor-1 (SDF-1), and other factors to promote endothelial cell proliferation and lumen formation, thereby improving local blood supply and oxygen delivery to the wound site (Xu et al., 2018; Zhu et al., 2016). Certain peptides, such as the laminin-mimetic peptide SIKVAV, can directly activate endothelial signaling pathways and enhance angiogenic capacity (Chen et al., 2015). Furthermore, during the collagen remodeling phase, peptide hydrogels regulate the balance between MMPs and TIMPs, facilitating the transition from type III to type I collagen and enhancing the mechanical strength of the tissue (Caley et al., 2015). It is noteworthy that peptide hydrogels can mimic the physicochemical properties of the ECM, providing a biomimetic microenvironment that supports cell migration, proliferation, and differentiation. For instance, peptides containing the Arg-Gly-Asp (RGD) sequence specifically recognize integrin receptors, enhancing the adhesion and migration of fibroblasts and keratinocytes to accelerate re-epithelialization and granulation tissue formation (Kemker et al., 2021). Moreover, peptide hydrogels exhibit excellent self-healing properties and injectability, allowing them to adapt to irregular wound shapes and maintain structural integrity within the dynamic wound microenvironment for sustained functionality (Chen et al., 2020). Their high molecular design flexibility enables responsiveness to microenvironmental changes such as pH, enzymes, or redox potential, facilitating intelligent drug release and precise modulation of cellular behaviors. Thus, through multi-target and multi-mechanistic synergistic actions, functionalized peptide hydrogels promote wound healing by enhancing cell adhesion, combating bacterial infection, regulating inflammation, stimulating angiogenesis, and supporting tissue remodeling, positioning them as next-generation intelligent wound dressings with broad application prospects.

## 3 Functionalized peptide hydrogels promote wound healing through dynamic stage-matching mechanisms

**FIGURE 2:**
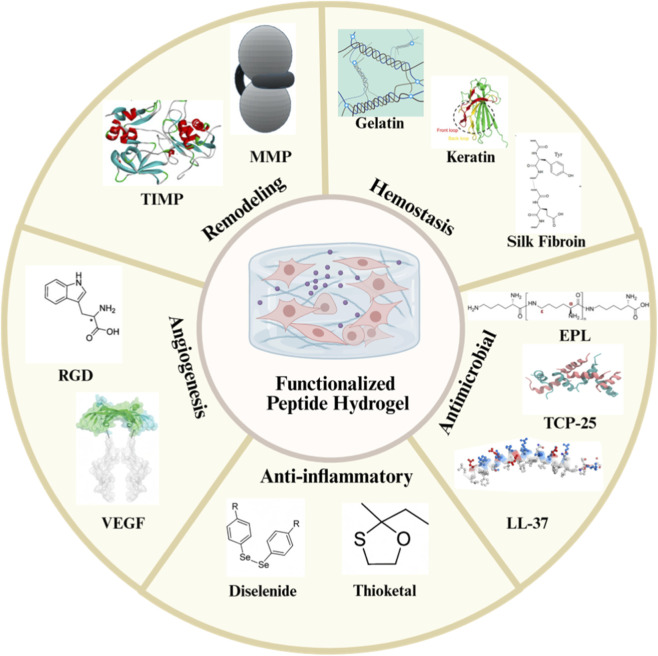
Functionalized hydrogels promote wound healing through dynamic stage-matching mechanisms (Zuev et al., 2024; Yu et al., 2022; Santi et al., 2021; Wang et al., 2024; Petruk et al., 2020; Martynowycz et al., 2019; Nogara et al., 2023; Ivanchenko et al., 2023; Wolkersdorfer et al., 2024; Kemker et al., 2021; Laronha et al., 2020).

Functionalized peptide hydrogels represent a highly promising strategy by employing stage-responsive design to dynamically coordinate multicellular behaviors—including hemostasis, antimicrobial defense, inflammation resolution, angiogenesis, and tissue remodeling—thereby synergistically promoting wound healing (Figure 2).

### 3.1 Hemostasis

Most hydrogels achieve hemostasis through adhesive effects to physically seal wounds (Rodrigues et al., 2019; Yang et al., 2024). While this simple mechanism is effective to some extent, improved outcomes can be achieved by dynamically matching the physiological mechanisms of hemostasis. This process can be modulated through two key pathways: platelet aggregation and activation of the coagulation cascade.

In 2012, Kumar et al. developed a series of nano-composite hydrogel bandages based on chitosan (CS) or chitin, demonstrating that cationic CS interacts with negatively charged blood cells to activate platelets and promote coagulation (Kumar et al., 2012). Subsequently, Fan et al. fabricated a CS/gelatin/PVA composite hydrogel and further elucidated the hemostatic mechanisms involving CS-induced platelet activation and coagulation factor production, in addition to physical sealing by the hydrogel (Fan et al., 2016). Further studies have shown that grafting positively charged components onto hydrogels enhances interactions with platelets, blood cells, and plasma fibronectin via electrostatic attraction, thereby inducing platelet activation and blood cell aggregation to promote coagulation. Besides CS, such cationic components include quaternary ammonium groups (Wang et al., 2019a; Venault et al., 2019), and cationic peptides (Yang et al., 2017). For example, gelatin exhibits strong water absorption and effective hemostatic activity. Studies have confirmed that it not only expands upon absorbing blood and tissue fluids to occlude bleeding wounds but also activates and aggregates platelets to facilitate coagulation (Huang et al., 2020; Han et al., 2021). Additionally, Rahmany et al. demonstrated that the loose, porous cross-linked network formed by keratin self-assembly can mimic the ECM, promoting platelet adhesion, activation, and the coagulation cascade (Rahmany et al., 2013; Wang et al., 2019b).

Wang et al. developed a quaternized hydroxyethyl cellulose/mesocellular silica foam hydrogel sponge (QHM) for hemostasis. The hydrophilic and highly absorbent QHM hydrogel attracts blood cells into its network. Besides the electrostatic interaction between quaternary ammonium groups and blood cells—which initiates clotting and platelet aggregation—an appropriate amount of mesoporous silica foam further activates factor XII (FXII). Thus, the QHM hydrogel significantly reduces *in vitro* plasma clotting time through these synergistic effects (Wang C. et al., 2019). Similarly, Lei et al. found that low-molecular-weight silk fibroin (SF) promotes activation of the coagulation cascade, accelerates fibrin formation, and strengthens blood clots to achieve hemostasis (Lei et al., 2016). Rahmany et al. also confirmed that keratin shortens plasma clotting lag time, promotes fibrinogen polymerization into fibrin, enhances coagulation, and reduces blood loss (Rahmany et al., 2013; Wang et al., 2019b). Furthermore, as a coagulation factor, calcium ions (Ca^2+^) promote blood clotting. Accordingly, Zhou et al. prepared an acetate chitosan/CaCO_3_ hydrogel in which H^+^ ions from acetate chitosan react with CaCO_3_ upon water absorption, releasing Ca^2+^ that not only strengthens the hydrogel network but also participates in hemostasis (Zhou et al., 2019).

Beyond dynamically matching the physiological process of hemostasis, functionalized peptide hydrogels can also deeply participate in subsequent healing phases. For instance, after platelet aggregation, growth factors and cytokines such as PDGF, transforming growth factor (TGF), epidermal growth factor (EGF), and insulin-like growth factor (IGF) are released—key mediators for subsequent healing stages (Rodrigues et al., 2019). This represents a significant advancement over mere physical sealing for hemostasis.

### 3.2 Antimicrobial

In the early wound phase, neutrophils and macrophages are recruited through various factors to eliminate pathogens by releasing antimicrobial granules (e.g., elastase, cathepsin G), forming NETs, and phagocytosis (Rodrigues et al., 2019).

As part of the innate epithelial chemical barrier, AMPs have recently garnered significant attention as promising broad-spectrum antibacterial agents against infections (Thakur et al., 2022). EPL, a natural polycationic homopolymer produced by *Streptomyces* albulus, has been widely investigated (Wang et al., 2024). Wang et al. developed a hydrogel co-loaded with EPL and MnO_2_ nanosheets, which demonstrated exceptional antibacterial efficacy against multidrug-resistant (MDR) bacteria, particularly *S. aureus* and MRSA, due to their synergistic effects (Wang et al., 2020). Liu et al. fabricated a self-healing bioadhesive hydrogel by blending modified hyaluronic acid (HA) and EPL. This inherently antibacterial hydrogel effectively eradicated *Escherichia coli* and *S. aureus* on wound surfaces and accelerated healing in an infected rat model (Liu et al., 2020). LL37, a human host defense peptide, is the only known cathelicidin in humans and plays a vital role in the innate immune system (Martynowycz et al., 2019). Yang et al. developed an LL-37/CS hydrogel dressing via physical mixing, which significantly inhibited the growth of *S. aureus in vitro* (Yang et al., 2020). Ma et al. designed a porcine small intestinal submucosa hydrogel incorporating LL37, which exhibited potent antibacterial properties (Ma et al., 2022). Other researchers developed a keratin-based hydrogel containing LL37, which not only promoted fibroblast proliferation, adhesion, and migration but also effectively eradicated both Gram-negative and Gram-positive bacteria and significantly enhanced microvascular density (Jelodari et al., 2023). TCP-25, a thrombin-derived C-terminal peptide consisting of 25 amino acids, has also shown therapeutic potential (Petruk et al., 2020). Puthia et al. developed a TCP-25-loaded hydrogel and thoroughly validated its antibacterial (against *S. aureus* and *Pseudomonas aeruginosa*) and anti-inflammatory activities in both mouse and partial-thickness porcine wound models. However, the stability of this hydrogel in human and porcine plasma requires further improvement, likely because TCP-25 does not bind to hydroxyethyl cellulose and is rapidly released into bodily fluids, resulting in a short half-life (Puthia et al., 2020).

Beyond these three widely studied AMPs, numerous other AMPs have been discovered and developed in various forms—such as nanoparticles, films, and hydrogels—to promote wound healing.

### 3.3 Anti-inflammatory

Following the antibacterial phase, persistent retention of neutrophils in the wound bed leads to excessive release of reactive oxygen species (ROS), activation of inflammatory signaling pathways, and further recruitment of immune cells, establishing a pro-inflammatory–ROS positive feedback loop (Rodrigues et al., 2019). In such cases, the impaired antioxidant system—including compromised function of superoxide dismutase and glutathione peroxidase—fails to clear ROS in a timely manner, resulting in sustained tissue damage. Therefore, controlling ROS levels and interrupting this feedback loop are central to anti-inflammatory strategies.

Given the strong context-dependence of ROS functions, there is a critical need to monitor and regulate ROS generation and concentration in wounds, which has motivated the development of tailored ROS-responsive biomaterials. Currently, ROS-responsive motifs can be classified into two major categories based on their mechanisms: The first category involves degradable motifs that undergo oxidative cleavage, leading to polymer chain dissociation and consequent changes in material properties—such as direct release of active ingredients or modulation of swelling behavior for indirect release. Commonly used motifs in this category include thioketal, diselenide, boronate ester, and aryl oxalate bonds. The second category relies on solubility-switching motifs that transition from hydrophobic to hydrophilic upon oxidation, thereby altering the swelling behavior of the material to achieve desired release profiles. Typical examples include thioether, ferrocene, and organochalcogen-based (e.g., selenium, tellurium) motifs. The development of diselenide-based ROS-responsive systems was pioneered by Ma and colleagues, who synthesized ROS-cleavable micelles capable of releasing glutathione upon hydrogen peroxide stimulation (Ma et al., 2010). Such designs have now been widely adopted. For instance, the research team led by Guo developed a gelatin-thioketal hydrogel that effectively reduced inflammation and promoted healing in diabetic wounds (Guo et al., 2022). Although numerous ROS-responsive materials have been explored, most current applications focus on fields such as oncology. Future efforts could leverage ROS-responsive mechanisms to deliver peptide-based therapeutics using hydrogels tailored for dynamic anti-inflammatory modulation in wound healing.

Furthermore, during the inflammatory phase, keratin has been shown to suppress the M1 phenotype while promoting polarization toward the M2 phenotype, resulting in reduced levels of pro-inflammatory cytokines (e.g., IL-1β, IL-6) and increased production of anti-inflammatory cytokines (e.g., IL-10) (Fearing and Van Dyke, 2014). This provides a mechanistic basis for developing anti-inflammatory stage-matched peptide hydrogels.

### 3.4 Angiogenesis

Angiogenesis is one of the most critical stages of wound healing, involving a complex interplay of cells (Rodrigues et al., 2019). Peptides most closely integrated with hydrogels can be classified into three categories: growth factor-related peptides (Xie et al., 2023), cell-adhesive peptides (Li et al., 2025b), and peptides that promote the macrophage polarization (Zhou et al., 2023; Li et al., 2025a).

During the pro-angiogenic process, growth factor-related peptides such as VEGF, PDGF, and bFGF play a fundamental role in promoting cellular proliferation. Wang et al. developed a VEGF-loaded hydrogel patch that achieves accelerated healing of diabetic wounds through rapid VEGF release in the high-glucose wound environment (Wang et al., 2025). In a similar approach, Zhang et al. developed a nano-responsive hydrogel loaded with PDGF, which also demonstrated efficacy in promoting the healing of diabetic wounds in animal studies (Zhang et al., 2025).

Cell-adhesive peptides primarily promote angiogenesis by facilitating the adhesion and migration of endothelial cells. The RGD sequence derived from fibrin not only provides critical support for endothelial cell migration and exerts selective chemotaxis (Montalbano et al., 2018), but also serves as an essential factor that synergistically drives neovascularization and granulation tissue formation (Dvorak et al., 1987; Heher et al., 2018). Zhu et al. demonstrated that the peptide YIGSR enhanced angiogenesis in a murine dorsal skin defect model, thereby accelerating wound healing (Li et al., 2025b). During the normal healing phase, pro-inflammatory M1 macrophages polarize toward an anti-inflammatory M2 phenotype as inflammation resolves (Galli et al., 2011). M2 macrophages release growth factors such as VEGF, which promotes vascular sprouting (Willenborg et al., 2012). Accordingly, Zhou et al. developed a hydrogel with dual antibacterial and anti-inflammatory activities; its loaded GL13L antimicrobial peptide not only combats pathogens but also promotes the transition from M1 to M2 macrophage polarization (Zhou et al., 2023). Similarly, Li et al. designed a gelatin methacryloyl hydrogel capable of sustained release of calcitonin gene-related peptide (CGRP), which was demonstrated both *in vitro* and *in vivo* to enhance macrophage polarization and angiogenesis (Li et al., 2025a).

These insights suggest that further elucidating the activation mechanisms and signaling pathways of other pro-angiogenic cells—such as through targeted agonism of specific receptors—may enable the development of more efficient and directly responsive hydrogels for dynamic stage-matched wound healing.

### 3.5 Remodeling

The remodeling phase is characterized by the regression of nascent vasculature, cyclical deposition and remodeling of the ECM, and the transformation of granulation tissue into scar tissue (Rodrigues et al., 2019). A key event in ECM remodeling is the targeted degradation of specific components by MMPs (Caley et al., 2015). During this process, *de novo* matrix synthesis slows significantly, leading to the gradual replacement of type III collagen with type I collagen within the granulation tissue (Caley et al., 2015). Upon ECM modification, TIMPs act to suppress MMP activity; an imbalance between TIMP and MMP expression can result in aberrant ECM remodeling and even contribute to chronic wound development (Gill et al., 2003; Taylor et al., 2000).

The MMP-responsive behavior of hydrogel systems is primarily mediated by proteins or peptides that serve as substrates for MMP cleavage. When exposed to sufficient MMP concentrations, these protease-sensitive motifs are hydrolyzed, leading to degradation of the hydrogel or drug-loaded microparticles and subsequent release of therapeutic agents. For example, Shao et al. encapsulated the pro-angiogenic drug deferoxamine (DFO) within gelatin microspheres. Incorporated into a chitosan-polyvinyl alcohol hydrogel matrix featuring ROS-responsive boronate ester bonds, these microspheres additionally responded to highly expressed MMP-9, enabling controlled DFO release. This system reduced oxidative stress in diabetic wounds while promoting angiogenesis, thereby creating a favorable microenvironment for remodeling and accelerating healing (Shao et al., 2023). Similarly, Zhang et al. developed a hydrogel composed of dopamine-functionalized oxidized hyaluronic acid, carboxymethyl chitosan, and collagen, which incorporated curcumin-loaded gelatin nanoparticles to enhance wound remodeling and repair (Zhang et al., 2023). Jeong et al. designed a hydrogel system based on functionalized hyaluronic acid and adamantane, where the latter was conjugated to AMPs via a cyclic peptide linker containing both MMP- and ROS-cleavable sequences. This dual-responsive strategy showed significant potential for treating chronic diabetic wounds (Jeong et al., 2023).

As demonstrated in these examples, the two most common strategies for incorporating MMP responsiveness into hydrogels are: (1) using gelatin as an MMP substrate—either as a matrix material or in drug/RNA-loaded microparticles; and (2) introducing MMP-sensitive peptide side chains or using MMP-cleavable cross-linkers within the hydrogel network. Both approaches effectively leverage MMP-responsive behavior to promote wound remodeling and healing, offering distinct advantages for treating chronic wounds such as diabetic ulcers. Furthermore, the design of hydrogels that act as dual sensors for MMP and TIMP to dynamically control therapeutic responses—thus enabling stage-specific interventions and promoting orderly tissue remodeling—represents a highly compelling strategy.

## 4 Summary and prospects

Functionalized peptide hydrogels represent a transformative advancement in wound care, moving beyond static barrier functions to actively orchestrate the dynamic healing process. By targeting multiple cellular behaviors and molecular pathways in a stage-specific manner, these hydrogels promote synergistic therapeutic outcomes, addressing the complexity of healing, particularly in challenging chronic wounds like diabetic ulcers. This review establishes a clear roadmap for developing “intelligent” wound dressings. The concept of designing materials that sense and respond to specific wound biomarkers (pH, enzymes, ROS) moves the field beyond one-size-fits-all solutions towards personalized, feedback-driven treatments. Future research should focus on developing sequentially multi-stimuli-responsive systems (e.g., synergistic sensing of MMP/ROS/pH), designing personalized peptide sequences, fabricating 3D-printed structural scaffolds, and engineering composite hydrogels loaded with cells or exosomes. Such advances are expected to accelerate the clinical translation and application of peptide hydrogels in wound management and precision regenerative medicine.
